# Couple-oriented prenatal HIV counseling for HIV primary prevention: an acceptability study

**DOI:** 10.1186/1471-2458-10-197

**Published:** 2010-04-19

**Authors:** Joanna Orne-Gliemann, Patrice T Tchendjou, Marija Miric, Mukta Gadgil, Maia Butsashvili, Fred Eboko, Eddy Perez-Then, Shrinivas Darak, Sanjeevani Kulkarni, George Kamkamidze, Eric Balestre, Annabel Desgrées du Loû, Francois Dabis

**Affiliations:** 1 Institut de Santé Publique Épidémiologie Développement (ISPED), Université Victor Segalen Bordeaux 2, Bordeaux, France; 2 Centre de recherche INSERM U897, Bordeaux, France; 3 Laboratoire d'Epidémiologie et de Santé Publique, Centre Pasteur du Cameroun, Yaoundé, Cameroun; 4 Centro Nacional de Investigaciones en Salud Materno Infantil, Santo Domingo, Dominican Republic; 5 Prayas Health Group, Pune, India; 6 Maternal and Child Care Union, Neoclinic, Tbilisi, Georgia; 7 Institut de Recherche pour le Développement, UMR 912 IRD-INSERM-U2 Marseille, France; 8 Institut de Recherche pour le Développement, UMR 196 CEPED, Paris, France

## Abstract

**Background:**

A large proportion of the 2.5 million new adult HIV infections that occurred worldwide in 2007 were in stable couples. Feasible and acceptable strategies to improve HIV prevention in a conjugal context are scarce. In the preparatory phase of the ANRS 12127 Prenahtest multi-site HIV prevention trial, we assessed the acceptability of couple-oriented post-test HIV counseling (COC) and men's involvement within prenatal care services, among pregnant women, male partners and health care workers in Cameroon, Dominican Republic, Georgia and India.

**Methods:**

Quantitative and qualitative research methods were used: direct observations of health services; in-depth interviews with women, men and health care workers; monitoring of the COC intervention and exit interviews with COC participants.

**Results:**

In-depth interviews conducted with 92 key informants across the four sites indicated that men rarely participated in antenatal care (ANC) services, mainly because these are traditionally and programmatically a woman's domain. However men's involvement was reported to be acceptable and needed in order to improve ANC and HIV prevention services. COC was considered by the respondents to be a feasible and acceptable strategy to actively encourage men to participate in prenatal HIV counseling and testing and overall in reproductive health services.

**Conclusions:**

One of the keys to men's involvement within prenatal HIV counseling and testing is the better understanding of couple relationships, attitudes and communication patterns between men and women, in terms of HIV and sexual and reproductive health; this conjugal context should be taken into account in the provision of quality prenatal HIV counseling, which aims at integrated PMTCT and primary prevention of HIV.

## Background

It is estimated that 2.5 million new HIV infections occurred worldwide in 2007 [1] and primary prevention remains a key intervention for mitigating the HIV/AIDS epidemic. Knowledge of one's HIV serostatus is critical not only for the prevention of mother-to-child transmission of HIV (PMTCT) and the provision of antiretroviral (ARV) treatment to HIV-infected adults, but also for the prevention of sexual transmission of HIV. Although the coverage of HIV counseling and testing services is increasing, it remains insufficient. Population-based surveys conducted in low- and middle-income countries between 2005 and 2007 estimated that a median of 10.9% of women and 10.3% of men had ever had an HIV test and received their test results [2]. In these countries, expanding PMTCT programs have increased the number of women tested during their pregnancy from 13% in 2004 to 21% in 2007 [2], but this is still largely inadequate. Further, HIV testing options for men are still limited to outpatient's consultations for Sexually Transmitted Infections (STIs), anonymous and voluntary HIV counseling and testing centers, and more recently male circumcision programs, and many opportunities are missed to involve men within PMTCT services. Thus worldwide, most men and women who live in couple with an HIV-infected partner are neither aware of his (her) HIV serostatus nor of their own. As a consequence, an HIV discordant situation is common in heterosexual couples throughout sub-Saharan Africa [3]. This pattern is also more and more frequent in other parts of the world like Asia, as the proportion of women newly infected with HIV increases [4]. There is thus an urgent need to define relevant, feasible and acceptable strategies to improve HIV prevention in a conjugal context.

In spite of repeated review and advocacy papers [5,6], very few scientific studies have investigated and published experiences of couple approaches to prenatal HIV counseling and testing, and PMTCT in general. In Zambia and Kenya, where pregnant women were offered individual or couple HIV counseling, in the absence of randomization, couple HIV counseling appeared to improve the acceptability of HIV testing, the uptake of ARV prophylaxis and the adherence to alternatives to prolonged and mixed breastfeeding [7,8]. Of note, the women who received couple counseling did not report an increased risk of adverse social events compared to individually-counseled women [8].

In addition, although the prevention of sexual transmission of HIV is an integral part of the WHO four-pronged approach to PMTCT [9], this is rarely emphasized in current PMTCT programs that usually focus on medical ARV interventions delivered to HIV-infected women. These missed opportunities for integrating the primary prevention of HIV and PMTCT should urgently be addressed. Identifying strategies that encourage the involvement of the male partner during the prenatal HIV counseling and testing process will most likely contribute to improving reproductive health and the prevention of HIV within the couple. This hypothesis, particularly relevant for settings where HIV prevalence rates are high, is also very relevant for low HIV prevalence settings where the benefits of PMTCT programs in terms of number of pediatric HIV infections prevented are *a priori *limited.

Community-based promotional strategies have recently been recommended in Zambian urban clinics to encourage couple HIV counseling and testing [10,11]. However there is a lack of data on the efficacy of simple and clinic-based behavioral interventions on the uptake of a couple approach to prenatal HIV counseling and testing. The ANRS 12127 Prenahtest trial aims to assess the impact of prenatal couple-oriented post-test HIV counseling (COC) on the incidence of partner HIV counseling and testing and couple HIV counseling, and on sexual, reproductive and HIV prevention behaviors. COC is a clinic-based behavioral intervention replacing standard post-test HIV counseling delivered to pregnant women. It provides the woman with personalized information as well as tools and strategies to actively involve her partner within the prenatal HIV counseling and testing process (Table 1). The framework of COC is based on available reference counseling modules [12], and partly inspired by the Health Belief Model [13].

**Table 1:** Content of couple-oriented post-test HIV counseling, as pilot-tested in the four study sites (2007-2008).

- Reminder of pre-test counseling messages	*Standard post-test counseling components*
- Announcement of HIV status	
- Standard counseling according to HIV status	
- Identification of the partner and discussion about the type of relationship	*Couple-oriented counseling components*
- Assessment of the level of couple communication regarding reproductive and HIV issues	
- Discussion about HIV status disclosure of HIV, partner HIV counseling and testing, couple HIV counseling	
- Provision of tools and strategies for the woman to address these issues with her partner	
- Anticipation of partners' possible negative reactions and strategies to overcome them	

The complete COC manual is available at http://etudes.isped.u-bordeaux2.fr/PRENAHTEST/PrenahtestInterventionManual_6May09.pdf

The Prenahtest HIV prevention trial is now ongoing in four low-to-medium HIV prevalence countries [14] and should be completed in 2011. This paper reports on the preparatory phase of the trial, conducted between June 2007 and January 2008. Our objective was to assess the acceptability of COC and of men's involvement in prenatal care services within different socio-cultural settings. Quantitative and qualitative research methods were used: direct observations of health services, in-depth interviews with key informants, field testing of the COC intervention and exit interviews with COC participants.

## Methods

### Study sites

The national adult HIV prevalence was estimated at 5.5% in 2004 in Cameroon [15], between 0.8 and 1.0% in 2007 in the Dominican Republic [16,17], around 0.1% in 2007 in Georgia [18] and reached 0.4% in 2007 in India [19]. Within each country, one urban tertiary hospital catering mainly for underprivileged populations was selected: Centre Mère et Enfant de la Fondation Chantal Biya in Yaounde (Cameroon), Hospital Materno-Infantil "San Lorenzo" de los Mina in Santo Domingo (Dominican Republic), Maternity Hospital N°5 in Tbilisi (Georgia) and Sane Guruji Hospital in Pune (India). Three of these four sites were part of a PMTCT implementation network sponsored by the Elizabeth Glaser Pediatric AIDS Foundation http://www.pedaids.org. In 2007 across the four study sites, 3422 women quarterly registered for their first prenatal care visit, ranging from 270 in the Georgian site to 1765 in the Dominican site. Over 90% of them received pre-test HIV counseling. HIV testing rates ranged from 50 to 100%. The overall post-test HIV counseling rate was 74%, varying from 30% in the Georgian site to 82% in the Dominican Republic.

### Baseline observations

A baseline assessment was conducted to describe existing prenatal HIV counseling services and to assess the context for COC integration. ANC consultations and education sessions, group and individual pre/post-test HIV counseling sessions were observed using structured observation sheets (n = 5 observations per service) (Table 2). The aim was to describe the logistical setting, the type of information provided by the health professional or counselor, and the relationship between the health care provider and the women. The observed consultations/sessions were purposively selected based on the socio-demographic characteristics of the patients: age, residence, number of children, nationality, marital status, level of education, among others. The observations were conducted by trained qualitative researchers.

**Table 2:** Overview of the research approaches used in the four study sites (2008).

	Cameroon	DR	Georgia	India	Total
	**n**	**n**	**n**	**n**	**n**

**Baseline observations**					
ANC consultation/education	5	6	5	5	21
Pre-test HIV counseling (individual)	NA	NA	10	5	15
Pre-test HIV counseling (group)	5	5	NA	5	15
Post-test HIV counseling (individual)	5	5	5	10	25
Post-test HIV counseling (group)	5	5	NA	NA	10

**Key informants in-depth interviews**	**22**	**20**	**28**	**22**	**92**
Senior medical/administrative staff	1	5	2	4	12
ANC nurse	0	0	2	0	2
PMTCT counselor	6	3	2	2	13
FP nurse/physician	1	2	2	1	6
Woman after pre-test HIV counseling	0	0	5	0	5
Woman after post-test HIV counseling	6	4	5	5	20
Woman after COC	6	4	5	5	20
Male partner	2	2	5	5	14

**Field testing of COC sessions**					
Pilot tested	30	30	30	30	120
Monitored	23	27	0	30	80
Observed	28	4	5	5	42

Cameroon = Centre Mère-Enfant (Yaounde), DR = Los Mina Hospital (Santo Domingo), Georgia = Maternity Hospital N°5 (Tbilisi), India = Sane Guruji Hospital (Pune).

ANC = antenatal care; COC = couple-oriented HIV counseling; NA = not applicable; FP = Family Planning

### Key informants in-depth interviews

In-depth interviews were conducted to assess local perceptions regarding HIV/AIDS and men's involvement within prenatal care, couple communication on HIV and the acceptability of COC (Table 2). Interview guides consisted in lists of topics to be discussed with each informant such as "attitudes of women/men/health professionals towards men's involvement within the local ANC/PMTCT process". Senior medical and administrative staff was interviewed as key decision-makers; nurses and PMTCT counselors were interviewed as key providers of PMTCT services; family planning nurses/physicians were interviewed as key providers of reproductive health services. Women having received standard post-test HIV counseling or COC were interviewed as potential beneficiaries of COC, and men accompanying their wives to ANC were interviewed as potential actors of the impact of COC. Women and men were purposively selected based on the same socio-demographic characteristics as listed above. Interviews were conducted by trained qualitative researchers and were tape-recorded.

### Field-testing of the COC intervention

Thirty COC sessions were pilot-tested in each site over a period of one to three months to assess the feasibility and acceptability of COC. One or two PMTCT counselor(s) were trained to deliver COC and relieved from standard HIV counseling duties. Pregnant women were informed and offered COC at pre-test HIV counseling or just before post-test HIV counseling. Women agreeing to COC were consecutively recruited until reaching the target of 30 women. Recruited women received their post-test session either on the day of recruitment or at a set appointment date a few days later. Out of the 120 women recruited, one from Cameroon was HIV-infected. COC sessions were monitored using a brief evaluation tool completed by the intervention counselor once the women had exited the room to document the content of the session, women's perceptions of their communication with their partner and wishes regarding the involvement of their partner within HIV counseling and testing were documented (Table 3). Finally, a sample of COC sessions was observed using the same selection criteria as for observations and in-depth interviews (Table 2).

**Table 3:** Women's reactions during the couple-oriented counseling monitored in the four study sites (2007-2008).

Women ....	Cameroon (n = 23)		DR (n = 27)		Georgia		India (n = 30)	
	
	n	%	n	%	n	%	n	%
Had ever talked about contraception with their partner	18	78.3	19	70.4	--	--	12	40.0
Had ever talked about sex with their partner	23	100	17	63.0	--	--	16	53.3
Had ever talked about HIV/AIDS with their partner	18	78.3	25	92.6	--	--	14	46.6
Had ever used condoms with their partner	18	78.3	10	37.0	--	--	8	26.6
Wished to disclose to their partner (first asked)	13	56.5	22	81.5	--	--	--	--
Wished to disclose to their partner (at the end of COC)	23	100	27	100	--	--	27	90.0
Wished to suggest HIV counseling and testing to their partner (first asked)	13	56.5	20	74.1	--	--	--	--
Wished to suggest HIV counseling and testing to their partner (at the end of COC)	23	100	27	100	--	--	28	93.3
Wished to receive couple HIV counseling (first asked)	20	87.0	25	92.6	--	--	27	90.0
Wished to receive couple HIV counseling (at the end of COC)	21	91.3	25	92.6	--	--	--	--
Wished to suggest couple HIV counseling to their partner (at the end of COC)	23	100	26	96.3	--	--	29	96.6

Cameroon = Centre Mère-Enfant (Yaounde), DR = Los Mina Hospital (Santo Domingo), Georgia = Maternity Hospital N°5 (Tbilisi), India = Sane Guruji Hospital (Pune).

Sessions observed between June 2007 and January 2008.

COC couple-oriented HIV counseling.

-- = Missing data.

### Ethics and support to participants

Our pre-trial assessment and trial protocols were approved by the ethics committee of our sponsor and main funder, the ANRS, as well as by the national and/or institutional ethics committees in the four study countries. Piloting committees composed of experts in the field of health, communication and social research, affiliated to the national Ministries of Health, academic institutions or local NGOs, were formed in each country to be consulted on the study monitoring and follow-up. An information leaflet was distributed to all participants. Informed consent was obtained from all health care workers, women and men interviewed or participating in the consultation or counseling sessions observed. For group sessions, all consents were collected on a single form. Financial incentives were not offered to the project participants. Free psychological support services, condoms and contraception until 18 months after delivery were routinely available or made available through the study for women upon request. Compensation for transportation costs of all additional visits to the health centre was provided to all participants.

### Data analysis and presentation of results

The COC data from the exit interviews and monitoring tool were entered and analyzed in an Excel database. In-depth interviews were recorded and transcribed in French (Cameroon), Marathi (India), Georgian (Georgia) and Spanish (Dominican Republic). Transcripts were translated into English, reviewed and analyzed separately by each study team using the content analysis [20] or the grounded theory approach [21]. Thematic synthesis [22] was used to compile, confront and organize site results according to specific topics derived directly from the text data and the interview guide, and guided by the general aims and research questions of the pre-trial phase.

The results presented in this paper combine the different sources of data collection and are organized according to four main themes which describe specific aspects of the acceptability of COC: the quality of existing prenatal HIV counseling, men's involvement in ANC, couple communication and HIV, and partner HIV testing.

## Results

### Existing prenatal HIV counseling services

The baseline observations documented many similarities across the four sites regarding existing prenatal HIV counseling services. For pre-test HIV counseling, group sessions were the norm, except in Georgia. Post-test HIV counseling was provided individually in three sites; however in the Dominican Republic, due to the workload and the lack of space, it was provided in groups except for HIV-infected women receiving additional individual sessions. No site provided post-test HIV counseling on the same day as HIV testing. The staff providing counseling had different professional and training backgrounds: counselors were nurses in Cameroon, obstetricians in Georgia, social workers in India, and peer counselors in the Dominican Republic site.

The content of routine prenatal HIV counseling provided in each site was comparable and addressed most aspects of HIV transmission and prevention and PMTCT in particular. However the importance of partner HIV testing and of the overall involvement of men within the prenatal HIV counseling and testing process was hardly discussed. Notably in Georgia, as post-test HIV counseling was mostly delivered by obstetricians at the next ANC visit, it often just included notifying the HIV test result.

All sites observed limited privacy for counseling and overall frequent interruptions by patients or other health care providers. This was reported to interfere with the woman's concentration and confidence to openly discuss private topics with the counselor. The observations also revealed the existence of "one-way relationships" between the counselor and the patient, whereby prenatal HIV counseling sessions were more like lectures than discussions. Most counselors did not assess women's understanding of the topics discussed. This was partly related to the counselor's skills but also to the lack of time made available for HIV counseling.

In spite of these logistical and quality constraints, the key informants reported positive perceptions of the HIV counseling provided in the study sites. Women, who in general preferred individual HIV counseling sessions, found the scientific information given by the counselor very useful. Health care workers declared that HIV counseling was a very effective preventive measure for HIV prevention, specifically by raising HIV/AIDS knowledge levels among the general population.

### Observed and desired involvement of men in ANC

If men's attendance at ANC services was reported to be acceptable by all informants, the practice greatly varied in the four study sites. In the Indian site, partners accompanied approximately 20% of ANC women. But in Cameroon, Georgia and the Dominican Republic, according to ANC nurses, only one or two couples were seen together in ANC every month. The respondents from Dominican Republic however underlined that an increasing number of men were now prepared to get involved in maternal and child health services, particularly in child care.

*"That has changed a lot in the country. Here we see in the consultations how the fathers walk around with the babies and diapers, and the bags next to the mother. Before - I am talking about 20 years ago - that could had been a reason for making fun of him, but at the moment no, (...), you see them, and it is a pride for those fathers" *(Dominican Republic, man, 51 years, doctor).

One of the main constraints for men's involvement in ANC was related to the organization of health services. Established practices observed across the four sites meant that men did not have access to ANC consulting rooms, that the space and time to care for men was insufficient and that the overall atmosphere in the ANC setting was not male-friendly.

*"If you see a man seated on that bench, the first thing they [health care workers]'ll do is to make him get up: 'Hey, friend, get up there, that bench is for pregnant women'. So, beside the fact that they don't come, the few of them who do come are not treated so 'well'..." *(Dominican Republic, woman, 32 years, doctor).

Furthermore, almost all respondents explained that reproductive health issues were not considered as a man's matter: maternity was the woman's domain and men attending ANC were perceived as being out of place. In India in particular, men who took interest in such activities along with their wives could be teased and labeled as "*sissies*".

"*The fact is that, even if a man wants to accompany his wife, the other family members, like his mother for instance, tell him that the things like pregnancy are all feminine affairs. Then he starts to see things in the same way, thinking that his mother, sister or aunt or sister-in-law will look into the matter. He then tends to wash his hands off.' *(India, man, 44 years, gynecologist).

In addition to these institutional and social barriers, most of the men interviewed mentioned that their professional activity and working hours meant that their presence in ANC was difficult or even impossible; this was also commonly reported by women and health care workers.

Among the respondents in the Dominican Republic, some men declared lacking interest in ANC and women would confirm this attitude by reporting low expectations regarding their partner's involvement and seeming passive and resigned.

*"I come alone because... I mean, if he was interested in coming with me he would have done it, since the beginning, when I first told him I was pregnant." *(Dominican Republic, woman, 20 years, standard post-test HIV counseling).

Finally, some providers in the Dominican Republic analyzed the absence of men's involvement in ANC as the expression of women's desire to preserve personal control over their reproductive health decisions.

In spite of these various observed constraints, all respondents declared that men's involvement within reproductive health was important and necessary. If actively and carefully explained about the goals of ANC and HIV counseling, male partners would be keener in participating in the prenatal HIV counseling and testing process. Respondents from the Dominican Republic suggested that in order to make sexual and reproductive health services attractive to men, promotion messages should be positively re-framed, from a perspective compatible with the perceived gender roles shared among the national popular sectors, and focused on men's protective role within the family.

### Couple communication on HIV

Women interviewed as key informants reported rather low couple communication about HIV. In Georgia, one woman explained that many people did not consider HIV relevant for them personally, as well as for the country's reality.

"*Once I tried to start conversation about this issue after the TV spot. He answered me that this disease is a problem of sex workers and drug users, neither you nor me are such persons." *(Georgia, woman, 27 years, pre-test HIV counseling).

Most women however declared that they avoided talking about safe sex, family planning and HIV/AIDS to their partner, because they did not expect him to be interested and they would feel embarrassed if they started the conversation about these issues. In Cameroon, some women declared that their partner might feel accused of infidelity if they initiated the discussion on STIs or HIV.

*"He would say to me: 'What are you getting to talking about this? You want to accuse me? How are we concerned about this? Or are you saying that I am the one going out and exposing our family?" *(Cameroon, woman, 32 years, COC).

When couple dialogue on HIV was reported, it usually took place after watching an advertisement on television or hearing from someone newly infected with HIV. And these discussions would stay general, indirect and very scanty.

Specific types of dialogue were reported in India and the Dominican Republic where some of the women interviewed mentioned that discussions on HIV and safe sex would arise during couple confrontations, in the context of other mutual questionings related to fidelity.

Finally, however, only in the Dominican Republic and in Cameroon would women report "*advising him [their partner]" *to protect himself in the sexual relationships he has "*on the streets*", i.e. with other partners. This practice is not culturally acceptable in India.

All the women interviewed after receiving COC declared that COC was likely to contribute to improve their couple communication about HIV. This counseling made them feel that HIV was not a taboo and that it could be easily the topic of conversation between the couple. More than 90% of the women beneficiaries declared willing to disclose and suggest HIV counseling and testing to their partner (*vs*. 50% willingness before COC) (Table 3).

Women also declared that they usually lacked the tools and words to talk to their partner and that the COC session helped them reach a better understanding about their relationships and overall couple communication.

*"She [the Intervention Counselor] explained to me that when you approach your partner to ask him for something or you want him to do something - the communication will not be very good if he's angry. She taught me how to do it and put it in practice, and it gave me good results" *(Dominican Republic, woman, 17 years, COC).

All women reported appreciating the opportunity to discuss personal issues and some of them also expressed, spontaneously, that their female friends and relatives should have the opportunity of receiving this type of post-test HIV counseling session.

### Perceived risk of HIV and attitudes regarding partner HIV testing

Among the women interviewed as key informants, most perceived themselves at risk of getting HIV infection from their husbands. They commonly referred to sexual mode of HIV transmission as "extramarital sexual relationship". Thus they considered that partner HIV testing was important to protect women's health and for PMTCT. In the Dominican Republic, some women in stable relationships believed that, "trusting" their partners or "trusting" that their partners would protect themselves with other women, keeps them safe from HIV and from the need for HIV testing. Overall however, women expressed that they would feel *"more at ease" *if they knew for sure that their partner was HIV-negative.

The health care workers interviewed reported that COC was very likely to contribute to "*involve a very vulnerable part of the population [in sexual and reproductive health services], which are the patients' [women's] partners" *(Dominican Republic, man, 51 years, doctor), to increase partner HIV testing and thus to improve HIV prevention.

In Georgia and India, women having received COC reported being confident that their partner would accept HIV testing. Most would convince their spouses by arguing on the importance for the family's and child's well being.

And indeed across the four sites, most men interviewed reacted positively to HIV testing by saying they would not mind doing the test, as they had never had any extramarital sexual relationship. The concept of HIV discordance however was not completely understood by the men interviewed. This was the case particularly in Cameroon where men did not see why they should be tested for HIV if their wife's result was known. In the Dominican Republic, the question about the willingness to be tested for HIV seemed potentially threatening and triggered anxious responses among some of the interviewed men, who repeatedly assured the interviewer that they were "*not afraid to be tested*" and that they "*have nothing to fear*", although they did not consider it was necessary for them to be tested for HIV.

During this preparatory phase of the trial, the actual number of men who returned for HIV testing after their wife/partner received COC was measured (Table 4). Overall, partner HIV testing rates reached 10 to 60% of women benefiting from COC over the study period, whereas routine rates of partner HIV testing, i.e. among women who did not receive COC during the study period, were estimated below 5% of women tested across the four study sites (overall rates: 2.7 vs 36.1%, p < 0.01).

**Table 4:** Use of partner HIV counseling and testing services after COC compared to baseline partner HIV testing rates in the four study sites (2008).

	Cameroon		DR		Georgia		India		Total	
	
	n	%	n	%	n	%	n	%	n	%
Baseline *	N = 394		N = 1049		N = 256		N = 1137		N = 2836	
Partner HIV testing*	3	0.7	41	3.9	3	1.2	30	2.6	77	**2.7**

Post-intervention**	N = 23				N = 30		N = 30		N = 83	
Partner individual counseling	9	39.1	--	--	9	30	0	0	18	21.7
Couple counseling	5	21.7	--	--	3	10	7	23.3	15	18.1
Partner HIV testing	14	60.8	--	--	11	36	5	16.6	30	**36.1**

Cameroon = Centre Mère-Enfant (Yaounde), DR = Los Mina Hospital (Santo Domingo), Georgia = Maternity Hospital N°5 (Tbilisi), India = Sane Guruji Hospital (Pune).

COC = couple-oriented HIV counseling

-- No data

* Estimated from local registers. Partners of "women tested over a 3-month period between June 2007 and January 2008".

** Partners of women having received COC.

## Discussion

In the preparatory phase of a multi-country HIV prevention trial, we assessed the acceptability of a simple clinic-based behavioral intervention aiming at a couple-approach to prenatal HIV counseling and testing.

Our first important result was that COC was perceived as acceptable by providers and beneficiaries. This couple-oriented prenatal HIV counseling, with a strengthened post-test session, was considered useful and beneficial by all key informants in the four study sites. This finding is very encouraging, in a general context where the need to identify both locally-adapted and simple, replicable interventions for HIV prevention is being advocated for [23]. We also documented a trend towards a positive impact of COC on couple communication regarding HIV, as a significant number of men returned for HIV counseling and testing after their wives received COC. Although no public health conclusion can be drawn from this small sample, these results are promising and confirm the need to continue with the trial phase.

However this acceptability assessment also highlighted that a certain form of status quo existed regarding the lack of men's involvement in ANC, maintained by the interaction of multidimensional factors involving institutional structures, service providers, pregnant women and men. The couple-oriented intervention is challenging the established gender norms as well as hospital practices and thus much time and effort will be needed to ensure men actually participate in prenatal HIV counseling and testing services. We indeed documented that the standard prenatal HIV counseling services provided in the four study sites did not take into account the woman's relationship with her partner. Yet the absence of disclosure is likely to expose women to difficulties in implementing the prevention messages she received such as using condoms or adopting alternatives to prolonged breastfeeding. By promoting COC, we are disrupting the established perceptions and practices of providing women-centered ANC and PMTCT services. The analysis of key informant interviews strongly suggest that one of the main constraints to men's involvement in ANC was the existence of gender norms by which ANC is labeled as a woman's domain. The fact that these reported perceptions were similar in the four study sites, which have different "socio-cultural" backgrounds in terms of couple relationships, sexual behavior, perceptions of HIV, or use of mother and child health services, confirm that these gender norms are probably not culture-specific. Our observations showed that, as in many other dimensions of reproductive health, the structural and conceptual basis of ANC and PMTCT programs has focused the attention on women. PMTCT services, integrated within ANC, maternity or infant immunization services, dedicated to women and children, have often excluded men de facto and are rarely male-friendly [24]. Introducing COC and promoting men's involvement within prenatal HIV counseling and testing during the trial phase has thus required several programmatic adjustments, such as securing individual counseling space for COC sessions, and providing access authorizations and sitting arrangements for men to facilitate the presence in ANC, among others. At a longer-term level, the routine delivery of COC may require that health administrators consider expanding ANC consultation hours or that health promoters revise the ANC educational material distributed to emphasize the need and challenges of a couple-oriented approach to HIV prevention, among others.

We also learnt that the existing prenatal HIV counseling services are likely to limit the personalization of prevention messages. We observed that the relationship between ANC nurses or HIV counselors and pregnant women was usually unidirectional, with patients used to listen to the health staff and unable to easily ask questions. Paternalistic provision of health care and social distance between patients and providers has been documented in southern Africa [25,26], however no data has been published so far in the field of HIV counseling. Yet, one of the keys to efficient post-test HIV counseling is for pregnant women to appropriate to themselves and identify with the counseling they receive. The pilot-testing of COC, during which women are encouraged to express their feelings and talk about intimate issues, provided an opportunity for each study site to reflect on the concept of HIV counseling, its objectives and delivery strategies. In the Georgian site, this pre-trial phase induced a deep change in the local perceptions of the importance of HIV counseling: prenatal HIV counseling is no longer provided in the study clinic by untrained obstetricians but by specialized HIV counselors. In the context of expanding provider-initiated HIV counseling strategies [27], less emphasis is put in delivering extensive pre-test HIV counseling. Within the ANC services adopting this "opt-out" approach to HIV testing, it remains crucial to ensure that informed consent for HIV testing is obtained in an ethical manner and that women tested for HIV are well supported in managing their HIV test results. And our study suggests that it is needed and acceptable to invest in providing quality post-test HIV counseling.

Because of the exploratory nature of this study phase, our results may have certain limitations. First, our key informants were purposively selected based on their professional or personal experience with ANC, PMTCT and FP services and thus their discourse may not be representative of the community viewpoints on this topic. Our sample of male partners is considerably lower than expected, as men were rarely present within the walls of our four study sites, and thus the voices of men may be underrepresented within our study results. Second, the international design and the necessary standardization of our data collection procedures and tools may have made the deeper understanding of the specificities of each site more difficult. And the need to translate and back-translate all tools, procedures and results between English and the four national languages, may have induced a loss of meaning in our analysis, an over-simplification of local expressions or concepts. Finally, it should be acknowledged that the study results were obtained through different data analysis techniques (content analysis and grounded theory). Conducting multi-country qualitative research can be complex [28]. In spite of these constraints and limitations, however, this multi-country design has provided a unique opportunity for exploring the acceptability of an innovative intervention in different settings and for strengthening international public health collaboration for HIV prevention as recently advocated [29]. We have highlighted that, within four different sites, over four continents, the existing assets and obstacles towards couple-oriented prenatal HIV counseling and testing were very similar, in spite of *a priori *very different socio-cultural, epidemiological and programmatic contexts.

## Conclusions

Overall, this pre-trial assessment suggested that one of the keys to men's involvement within prenatal HIV counseling and testing is the better understanding of couple relationships, attitudes and communication patterns between men and women, in terms of HIV and sexual and reproductive health. This conjugal context needs to be taken into account in order to provide quality prenatal HIV counseling, which aims at integrated PMTCT and primary prevention of HIV [30].

During the trial phase, we will use now a combination of qualitative and quantitative research methods to assess the impact of COC in comparison to standard post-test HIV counseling and monitor couple communication regarding HIV and other sexual and reproductive health issues from pre-test HIV counseling until 15 months after delivery. We hypothesize and hope to demonstrate that simple and efficient couple-oriented interventions are likely to be a keystone to successful combined HIV prevention [31].

## Competing interests

The authors declare that they have no competing interests.

## Authors' contributions

JOG conceived the study, coordinated the overall study implementation and data analysis and drafted the manuscript. PT supervised the design of the study, supervised the study implementation and data analysis for the Cameroon site. MM participated in the design of the study, supervised the study implementation and data analysis for the Dominican Republic site. MG conducted the field work and data analysis in the Indian site. MB participated in the Georgia site. FE participated in the design of the study and in the study supervision and data analysis for the Cameroon site. EPT participated in the design of the study, supervised the study implementation and data analysis for the Dominican Republic site. SD participated in the design of the study, supervised the study implementation and data analysis for the Indian site. SK participated in the design of the study and supervised the study implementation for the Indian site. GK participated in the design of the study for the Georgian site. EB participated in the data analysis. ADL supervised the design of the study. FD supervised the design of the study. All authors helped to draft the manuscript, read and approved the final manuscript.

## Pre-publication history

The pre-publication history for this paper can be accessed here:

http://www.biomedcentral.com/1471-2458/10/197/prepub
